# Knowledge of Health Benefits of Fruits, Vegetables, and Antioxidants, Propensity to Sustainability and Adherence to the Mediterranean Diet: An Interrelated Evaluation

**DOI:** 10.3390/nu18101490

**Published:** 2026-05-07

**Authors:** Patrizia Calella, Mario Siervo, Concetta Paola Pelullo, Fabrizio Liguori, Giuliana Valerio, Giorgio Liguori, Francesca Gallè

**Affiliations:** 1 Saint Camillus International University of Health and Medical Sciences (UniCamillus), 00131 Rome, Italy; 2 Dementia Centre of Excellence, School of Population Health, Curtin University, Perth 6102, Australia; 3 Department of Medical, Movement and Wellbeing Sciences, University of Naples “Parthenope”, 80133 Naples, Italy

**Keywords:** nutrition knowledge, fruit and vegetable consumption, antioxidants, Mediterranean diet adherence, sustainable food choices, public health nutrition

## Abstract

**Background/Objectives**: Beyond health benefits, plant-based diets are increasingly recognized for their contribution to environmental sustainability. This study aimed to evaluate knowledge of the health benefits of fruits, vegetables, and antioxidants, adherence to the Mediterranean Diet (MD), and propensity toward sustainable food purchasing in an adult population from Southern Italy, and to explore the relationships among these factors. **Methods**: A cross-sectional online survey was conducted among 311 adults living in the Campania region. Data were collected on knowledge of fruit and vegetable health benefits, antioxidant knowledge, adherence to the MD assessed using the Medi-Lite score, and attitudes toward sustainable food purchasing. Differences were examined according to gender, age, and educational level. Pearson’s correlation analyses and multiple logistic regression models were used to investigate associations between nutrition knowledge, dietary adherence, and sustainability-related behaviors. **Results**: Participants showed a medium–high level of knowledge regarding the health benefits of fruits and vegetables, while knowledge of antioxidants was moderate and significantly higher among older adults. Overall adherence to the MD was moderate, with lower consumption of vegetables, legumes, and fish. Most participants reported limited attention to sustainability when purchasing food. Both knowledge of fruit and vegetable health benefits and antioxidant knowledge were positively associated with sustainable food purchasing. In regression analyses, lower nutrition knowledge was independently associated with reduced odds of sustainable purchasing, whereas adherence to the MD was not a significant predictor. **Conclusions**: Although nutrition knowledge was generally adequate, its translation into sustainable food choices remained limited. These findings support the need for integrated public health strategies to encourage healthier and more environmentally responsible dietary behaviors.

## 1. Introduction

Regular consumption of fruits and vegetables is a cornerstone of healthy diet and plays a pivotal role in the prevention and management of various chronic diseases. These foods are rich in essential nutrients, including vitamins, minerals, and dietary fiber, and are a primary source of antioxidants, which help combat oxidative stress and reduce inflammation in the body [1]. Numerous studies have shown that a diet high in fruits and vegetables is associated with a reduced risk of cardiovascular diseases, certain cancers, and other health conditions [2]. These protective effects are attributed to the high content of dietary fiber, potassium, and antioxidants such as flavonoids and carotenoids, which help reduce blood pressure, improve vascular function, and decrease inflammation. The World Cancer Research Fund and the American Institute for Cancer Research highlight that non-starchy vegetables and fruits may protect against several types of cancer, including those of the mouth, pharynx, larynx, esophagus, and stomach [3]. Specific fruits and vegetables, such as cruciferous vegetables and citrus fruits, have strong protective effects due to their high levels of glucosinolates and vitamin C [4].

As a matter of fact, fruits and vegetables contribute to overall dietary quality and are fundamental to dietary patterns such as the Mediterranean Diet (MD), which is renowned for its health-promoting properties [5].

The World Health Organization (WHO) recommends a minimum intake of 400 g of fruits and vegetables per day as part of a healthy diet to reduce the risk of non-communicable diseases (NCDs) [6]. Recommendations highlight that adequate intake of these foods is crucial for maintaining a healthy body weight and preventing micronutrient deficiencies.

Incorporating more fruits and vegetables into one’s diet not only offers health benefits but also contributes to environmental sustainability. Plant-based diets such as MD are associated with lower greenhouse gas emissions, reduced land use, and decreased water footprint (i.e., water use in food production) compared to diets high in animal products [7]. Additionally, consuming locally grown and seasonal produce minimizes the environmental impact associated with transportation and storage, further promoting sustainability [8].

However, despite these benefits, global consumption levels of fruits and vegetables are often below the recommended amounts, particularly in low- and middle-income countries [9]. Understanding consumer knowledge about the effects of fruit and vegetable consumption is fundamental to design effective policies of healthy diet promotion. Moreover, as the global population grows and environmental concerns become more pressing, there is an increasing need to explore people’s propensity towards sustainable food.

This study aims to evaluate knowledge regarding the health effects of fruits, vegetables and antioxidant components, adherence to the MD, and knowledge and attitudes toward sustainable products in a sample of adults from the Campania region, South Italy, assessing possible differences based on their gender, age and educational level. A secondary aim is to explore whether adherence to the MD is associated with a greater propensity to purchase sustainable products.

## 2. Materials and Methods

### 2.1. Study Design and Participants

A cross-sectional study design was adopted. Data were collected between April and September 2024. The study targeted adult participants from diverse social and demographic backgrounds, aiming to gather data from a broad spectrum of the population. Participation in the study was entirely voluntary, and inclusion criteria were limited to the ability to read and understand the informed consent and to independently complete the online questionnaire.

Participants were recruited through a chain mailing list distributed across various networks, facilitating access to the general population. A total of 349 individuals accessed the questionnaire. After data cleaning, 38 responses were excluded due to incompleteness, inconsistent, or duplicate answers, resulting in a final sample of 311 participants included in the analysis. Although a formal a priori sample size calculation was not performed due to the exploratory nature of the study and the voluntary recruitment strategy, a post hoc estimation was conducted to assess the adequacy of the sample size. Based on previous literature reporting the prevalence of adherence to the MD [10], and assuming a 95% confidence level and a margin of error of 5%, the estimated required sample size was approximately 329 participants. Therefore, the final sample included in the present study (*n =* 311) can be considered adequate for the purposes of the analysis.

The anonymous structured questionnaire was available online, allowing participants to complete it at their convenience. To ensure data quality and reliability, questionnaires with incomplete, random or repeated answers were excluded from the final analysis.

This approach ensured a wide reach while maintaining the integrity of the data collection process. By allowing voluntary participation and providing clear information about the study goals, the recruitment strategy emphasized inclusivity while adhering to ethical research principles.

The study was conducted in accordance with the Declaration of Helsinki and was approved by the Ethics Committee of the University of Campania “Luigi Vanvitelli” (approval no. 28480/2022). All participants provided informed consent before completing the questionnaire.

### 2.2. Questionnaires

The questionnaire consisted of four sections designed to assess different aspects of participants’ characteristics, knowledge, and attitudes.

The first section collected demographic and anthropometric data, including age, gender, education level, occupation, weight and height. Age and occupation were collected through open-ended questions. Education level was assessed using three categories according to the Italian education system: primary/lower secondary education, high school diploma, and university degree or higher. Participants were also asked to self-report their height and weight, which were used to calculate Body Mass Index (BMI) as weight in kilograms divided by height in meters squared (kg/m^2^). Additionally, participants were asked whether they were currently following a specific dietary plan through a dichotomous question (“yes”/”no”).

The second section aimed to assess participants’ knowledge of the health effects of fruit and vegetable consumption. The questionnaire was based on the instrument developed by Le Turc et al. [11] and included the same 11 items assessing knowledge of the health effects of fruit and vegetable consumption. The items were translated into Italian and slightly adapted linguistically to ensure clarity and comprehension, while preserving the original meaning and structure of the statements. Participants were asked to indicate their level of agreement using a five-point Likert scale: “Strongly disagree,” “Disagree,” “Neither agree nor disagree,” “Agree,” and “Strongly agree.”

Responses were numerically coded on a standardized scale ranging from −2 to +2, where higher values indicated greater knowledge. Statements corresponding to incorrect assertions (items 1, 3, and 7) were reverse-coded to ensure consistency in the direction of scoring, as described in the original study [11]. Responses indicating neutrality (“Neither agree nor disagree”) were excluded from the scoring procedure to avoid reducing the discriminative capacity of the knowledge assessment and to limit central tendency and acquiescence bias, as suggested in previous survey methodology research [12]. This approach ensured that the resulting score reflected a clear orientation toward correct or incorrect knowledge, thereby improving the interpretability of the construct. Higher scores were interpreted as a greater ability to correctly identify evidence-based health effects of fruit and vegetable consumption and to distinguish them from common misconceptions. For each participant, an overall knowledge score was calculated as the mean of all item scores. Based on this average score, knowledge levels were categorized into four classes: very low knowledge (−2 to −1), low knowledge (−1 to 0), high knowledge (0 to 1), and very high knowledge (1 to 2), in accordance with the methodology proposed by Le Turc et al. [11]. These predefined score ranges were used to ensure a standardized interpretation of knowledge levels and to allow comparability with previous studies using the same instrument.

The third section assessed participants’ knowledge of antioxidant compounds using a questionnaire derived from a previous study on consumers’ knowledge of antioxidants [13]. The instrument included 14 statements addressing the role of antioxidants in health, including their effects on cancer prevention, aging processes, immune function, metabolic regulation, and common misconceptions related to their sources and use. The items were translated into Italian while preserving the original meaning and structure of the statements. Each item was presented in a three-option format (“True”, “False”, and “I don’t know”), consistent with the original study. For scoring, correct answers were assigned a value of 1, whereas incorrect and “I don’t know” responses were scored as 0. A total knowledge score was calculated by summing all responses, with higher scores indicating greater knowledge of antioxidant compounds. The total score ranged from 0 to 14, with higher values reflecting a greater ability to correctly identify the biological roles of antioxidants and to distinguish scientifically supported information from common misconceptions. For interpretative purposes, knowledge levels were categorized into three groups based on the percentage of correct answers: low (<50% of correct responses), moderate (50–75%), and high (>75%), in line with commonly used approaches in nutrition knowledge assessment studies. These categories were introduced to provide a standardized interpretation of the knowledge construct and to facilitate comparison with previous research [13].

The fourth section evaluated participants’ adherence to the MD, using the Medi-Lite score, a literature-based and validated instrument developed by Sofi et al. [14]. The questionnaire evaluates the consumption of nine food groups: fruits, vegetables, cereals, legumes, fish, meat and meat products, dairy products, alcohol, and olive oil. For each food group, participants reported their habitual intake based on predefined portion sizes and frequency categories (daily or weekly consumption), as specified in the original validation study. For example, fruit consumption was categorized as <1 portion/day, 1–2 portions/day, or >2 portions/day, while fish intake was categorized as <1 portion/week, 1–2.5 portions/week, or >2.5 portions/week. Similarly, dairy product consumption was classified as <1 portion/day, 1–1.5 portions/day, or >1.5 portions/day. Alcohol intake was categorized based on daily alcohol units (1 unit ≈ 12 g of alcohol), with <1 unit/day, 1–2 units/day, and >2 units/day as reference categories. Olive oil consumption was assessed based on frequency of use (occasional, frequent, or regular), reflecting its role as the main source of dietary fat in the MD. For food groups typical of the MD (fruits, vegetables, cereals, legumes, and fish), scores of 2, 1, and 0 were assigned to high, medium, and low consumption levels, respectively. Conversely, for food groups not typical of the MD (meat and dairy products), scoring was reversed, assigning 2 points to low consumption, 1 point to moderate consumption, and 0 points to high consumption. For alcohol intake, moderate consumption was assigned the highest score, while both low and high consumption received lower scores, according to the original scoring system. Olive oil consumption was scored based on frequency of use as the main culinary fat: 2 points for regular use (daily use as the primary added fat), 1 point for frequent use, and 0 points for occasional use. The total Medi-Lite score was calculated as the sum of all items and ranged from 0 (lowest adherence) to 18 (highest adherence), with higher scores indicating greater adherence to the Mediterranean dietary pattern. Adherence to the MD was also categorized into three levels (low ≤ 9, moderate 10–11, high ≥ 12), based on previously proposed cut-offs [14].

Finally, a single-item measure was included to assess participants’ propensity to consider sustainability when purchasing food products. Participants were asked: “Do you purchase food products based on their sustainability?” Responses were recorded using a five-point ordinal scale: “Always,” “Often,” “Sometimes,” “Rarely,” and “Never.” This item was specifically developed for the purposes of the present study to provide an exploratory assessment of sustainability-oriented purchasing behavior. Although single-item measures may have limitations, they are commonly used in survey research to capture general behavioral tendencies.

### 2.3. Data Analysis

Descriptive statistics, including means, standard deviations, frequencies, and percentages, were calculated to provide an overview of the sample characteristics. Demographic variables included age, gender and educational level. BMI was calculated from self-reported height and weight data.

Differences in knowledge scores, adherence to the MD, and sustainability-related behavior were evaluated according to gender, age category, and educational level using independent samples t-tests and one-way ANOVA, as appropriate. Additionally, comparisons between age groups (young adults 18–34 years old, adults 35–64 and older adults ≥ 65) were included to provide further insights.

The sustainability variable, originally measured on an ordinal scale, was treated as a continuous variable for descriptive and comparative analyses, as commonly done in survey-based research. For regression analyses, it was dichotomized to distinguish between low (never/rarely) and higher (occasionally/often) propensity toward sustainable purchasing.

Pearson’s correlation coefficients were calculated to examine the relationships between adherence to the MD (as measured by the Medi-Lite Score) and participants’ knowledge of antioxidants, the health benefits of fruits and vegetables, and attitude to sustainability.

Furthermore, binary logistic regressions were performed to identify predictors and correlates of the propensity to purchase sustainable foods. The outcome variable was dichotomized as follows: 0 = never/rarely and 1 = occasionally/often. Independent variables entered in the model were gender (0 = male, 1 = female), age (1 = lower than median value, 2 = higher than median value), educational levels (1 = university degree, 0 = all other education levels) adherence to the MD (1= lower than median value, 2 = higher than median value), knowledge of the health effects of consuming fruits and vegetables (1= lower than median value, 2 = higher than median value), and knowledge of antioxidant components (1= lower than median value, 2 = higher than median value). Odds ratios (OR) with 95% confidence intervals (95%CI) were reported to quantify these associations.

The data analysis was conducted using IBM SPSS Statistics for Windows, version 29 (IBM Corp., Armonk, NY, USA), and the significance level was set at *p* < 0.05. This analytical approach allowed for a comprehensive assessment of the differences and relationships among the key variables of interest within the study.

## 3. Results

The main characteristics of the study population are reported in Table 1. The sample consisted of 311 participants, including 131 males and 180 females, with a mean age of 45.83 ± 15.16 years; young adults represented 23.8% of the sample, while adults constituted the majority, accounting for 65.3%, and older adults comprised 10.9% of the sample. Males showed a significantly higher BMI compared to females (*p* = 0.022). A higher proportion of females reported currently following a diet compared to males, although this difference did not reach statistical significance (*p* = 0.069).

The results related to participants’ knowledge, attitudes, and adherence to the MD are summarized in Table 2.

Regarding knowledge of the health effects of fruit and vegetable consumption, the overall average score was 0.88 ± 0.74, indicating a medium-high level of knowledge. Based on the predefined categories, only 0.3% of participants showed a very low level of knowledge, approximately 15% had a low level, 35% a moderate level, and 49% a high level of knowledge. Although the majority of participants correctly recognized the beneficial effects of fruit and vegetable consumption, some misconceptions and uncertainties emerged. For instance, about 25% of participants did not agree or were uncertain that regular consumption of fruits and vegetables is associated with a longer and healthier life. Similarly, only 47% correctly identified fruits and vegetables as a good source of dietary fiber, while a substantial proportion reported uncertainty. Misconceptions were also observed regarding the iron content of vegetables and their role in anemia. Fruit and vegetable knowledge scores did not differ significantly according to sex, age group, or educational level.

With respect to knowledge of antioxidants compounds, the mean score was 7.88 ± 3.93 out of a maximum possible score of 14, indicating a medium level of knowledge.

Several gaps in knowledge were identified, particularly concerning the physiological role and metabolism of antioxidants. A large proportion of participants were uncertain about the factors influencing antioxidant requirements, the possibility that antioxidants can be both natural and synthetic, and the potential harmful effects of excessive intake.

No significant differences were observed between males and females nor considering the educational levels, but a significant difference was encountered considering the age category, (*p* = 0.012), older adults obtained a significant higher score than adults and young adults.

Finally, the mean adherence to the Mediterranean Diet, assessed using the Medi-Lite score, was 9.51 ± 2.12 (range 0–18). Based on previously proposed cut-offs (low ≤ 9, moderate 10–11, high ≥ 12), this value indicates an overall moderate level of adherence in the study population [14]. Olive oil stands out as the food group with the highest level of optimal adherence, followed by dairy and meat. In contrast, poor adherence is most evident in vegetables, legumes, and fish, with 34%, 37%, and 38% of participants respectively, reported consumption levels corresponding to the lowest category (i.e., less than one serving per day for vegetables and less than one serving per week for fish). Scores did not differ significantly according to sex, age group, or educational level.

Regarding sustainability-related purchasing behavior, more than half of the participants (57%) reported never considering sustainability when buying food products, while only a small proportion reported doing so frequently.

The correlation analysis reveals several significant findings that highlight the interconnections between knowledge of the health effects of fruits and vegetables and antioxidant knowledge. Knowledge of the health effects of fruits and vegetables was also significantly correlated with sustainable food purchasing. Antioxidant knowledge showed a significant correlation with sustainable food purchasing as well. A weak but statistically significant correlation was found between the Medi-Lite score and sustainable food purchasing. No significant correlation was found between antioxidant knowledge and the Medi-Lite score (Table 3).

Table 4 reports the results of a binary logistic regression analysis examining the factors associated with a higher propensity to purchase sustainable food products (occasionally/often vs. never/rarely). Among the variables included, both lower knowledge of the health benefits of fruits and vegetables and lower antioxidant knowledge were significantly associated with reduced odds of purchasing sustainable products.

No statistically significant associations were found for age, gender, educational level and adherence to the MD.

## 4. Discussion

This study aimed to explore participants’ knowledge of the health benefits of fruits, vegetables, antioxidants, and propensity towards sustainable food. The findings showed a good knowledge of fruit and vegetable health benefits, and limited understanding of antioxidants in the sample, though older adults performed better. Awareness of sustainability was generally low, influenced by education level, and most participants reported minimal attention to sustainability in their purchasing habits.

Furthermore, a moderate adherence to the MD was found in our sample. Several studies support the findings of the present research, indicating a moderate adherence to the MD among Italian citizens, particularly in central and southern regions. A study conducted by the University of Florence on a population with a comparable age range reported similar levels of adherence [15]. This trend is further confirmed by a large-scale Italian study carried out between 2019 and 2022, which involved over 10,000 adults and documented an overall good adherence to the MD [16]. However, the same study also revealed a gradual decline in adherence over time, characterized by a reduced consumption of key MD components such as vegetables, legumes, and fish, and an increased intake of animal products like red meat and cheese [16]. In our sample, a similar pattern emerged when analyzing adherence by food group. Optimal adherence was observed for olive oil, while lower adherence was found for vegetables, legumes, and fish, key elements of the traditional MD. These results mirror those reported in the ARIANNA study, which also noted a shift in dietary habits among Italians away from foundational MD components [17].

Participants exhibited a medium-to-high level of knowledge regarding the health benefits of fruits and vegetables, confirming a general awareness of their role in disease prevention and overall well-being. However, some misconceptions about specific nutrient contents, such as the role of certain vegetables in treating anemia or the fiber content of fruits, persisted, indicating areas where nutritional education should be improved. These findings are in line with a study by Grosso et al. conducted in Italy showing that, although most participants recognized the importance of fruits and vegetables in reducing chronic disease risk, only a small proportion of them could accurately identify specific health-promoting components such as fiber, vitamins, and phytochemicals [18]. Similarly, studies in the UK highlighted that while public awareness campaigns had improved general knowledge, gaps remained in understanding the variety and frequency of consumption needed for tangible health benefits [19,20].

Regarding antioxidant knowledge, our findings revealed a moderate level across the sample, with older adults scoring significantly higher than young adults and adults. This age-related difference might reflect greater exposure to health education over time or higher engagement with health-related content among older populations. These results echo those of the NutriNet-Santé study, where older participants were found to have more accurate and nuanced knowledge of antioxidant mechanisms and their relevance in preventing oxidative stress-related conditions [21].

Despite the fair level of knowledge about fruits, vegetables, and antioxidants, it is important to highlight a critical gap between knowledge and practice. While awareness is a necessary first step, it does not automatically lead to behavioral change. As observed in our study and supported by literature, the habit of consuming the recommended five portions of fruits and vegetables per day, as advised by the WHO and embedded in the MD model, has significantly declined in recent years among the Italian population [22,23]. This mismatch between knowledge and dietary behavior may be attributed to several factors; according to a systematic review by Munt et al., barriers such as time constraints, cost, taste preferences, and limited access to fresh produce can inhibit individuals from translating knowledge into consistent dietary habits [24]. Additionally, cultural and generational shifts in eating patterns, especially among younger adults, may further contribute to the erosion of traditional diet models such as the MD, despite general acknowledgment of their health benefits [24].

Interestingly, while adherence to the MD was positively associated with sustainable food purchasing behavior in the correlation analysis, this association was not confirmed in the logistic regression model. This discrepancy may be explained by several factors. First, the regression model accounted for multiple variables simultaneously, including knowledge of fruit and vegetable health effects and antioxidant knowledge, which were strongly associated with both MD adherence and sustainability-related behaviors. This adjustment may have attenuated the independent contribution of dietary adherence. Second, potential collinearity among predictors, particularly between knowledge variables and dietary patterns, may have further reduced the ability to detect independent associations. Finally, the use of a dichotomized outcome variable for sustainable purchasing behavior may have reduced the sensitivity of the analysis compared to the continuous approach used in the correlation analysis. These findings suggest that the relationship between MD adherence and sustainability-related behaviors is complex and may be partially mediated by knowledge-related factors.

Regarding sustainability, most participants in our study reported limited awareness and purchasing habits aligned with sustainability, but those with higher education demonstrated a better understanding of the concept and its definitions. This aligns with the results of another Italian study highlighting that food sustainability was strongly influenced by consumers’ perceptions and values, showing that higher levels of knowledge and attitudes towards food sustainability, particularly among women, were significant predictors of sustainable behaviors [25].

The present findings reveal a generally low level of engagement with sustainability, with the majority of participants reporting that they either never or only rarely consider sustainability when selecting food products. Only a small proportion reported frequently factoring sustainability into their decisions. However, this finding should be interpreted with caution, as sustainability-oriented purchasing behavior was assessed using a single-item, non-validated measure, which may not fully capture the complexity and multidimensional nature of sustainability-related attitudes and behaviors.

Though, this trend underscores a persistent gap between sustainability awareness and the actual implementation of sustainable behaviors. A similar study indicated that Italian consumers’ perceptions of food sustainability do not consistently translate into sustainable purchasing behaviors, highlighting a disconnect between awareness and action [26]. A recent cross-country study conducted on 6500 consumers, revealed that consumer behavior varies significantly depending on age, gender, and country of origin. For instance, Chinese consumers showed strong interest in sustainable certifications but reported generally higher consumption across food categories. In contrast, Danish adults and older individuals reported lower consumption of cheese, meat, fruits, and vegetables, accompanied by reduced interest in sustainability certifications [27]. Further insights are provided by a recent cross-sectional study involving 1000 individuals, which examined the relationship between health consciousness and pro-environmental behavior. Although sustainable food consumption was not among the most commonly practiced behaviors, lagging behind actions such as energy saving and recycling, the study found that individuals who were more attentive to their health were significantly more likely to engage in various sustainable behaviors [28].

Moreover, the observed correlations between knowledge of fruits and vegetables, antioxidant knowledge, and sustainability awareness reinforce the notion that health and environmental awareness are interrelated. Participants with greater understanding of the health benefits of plant-based foods were also more likely to be informed about sustainability concepts and to exhibit a stronger propensity for sustainable food choices. This finding supports previous research suggesting that health-oriented individuals are more receptive to environmental considerations, as shown in a study in which individuals adhering to a health-conscious diet were more likely to engage in pro-environmental behaviors compared to those in other groups [29]. Importantly, the health-conscious and mainstream diets were associated with lower greenhouse gas emissions than the traditional diet, mainly due to reduced energy intake rather than the specific food composition.

MD is a dietary model that integrates both nutritional adequacy and environmental sustainability. Indeed, the Mediterranean dietary pattern is characterized by a high consumption of plant-based foods, limited intake of animal products, and a preference for seasonal and locally sourced ingredients, all of which contribute to a reduced environmental footprint [30]. Several studies have demonstrated that adherence to the MD is associated with lower greenhouse gas emissions, reduced land use, and decreased water consumption compared to Western dietary patterns. Therefore, the MD represents a paradigmatic example of how healthy dietary patterns can simultaneously support human health and environmental sustainability, strengthening its role as a key framework for public health and sustainability-oriented interventions [31].

In addition to its well-established environmental sustainability, the MD may also confer health benefits through plant-specific adaptive mechanisms. Plants typical of Mediterranean ecosystems are exposed to environmental stressors such as drought and high solar radiation, which can enhance the accumulation of bioactive compounds, including polyphenols and other antioxidants. These compounds may contribute to the health-promoting effects of the MD, linking environmental adaptation to nutritional quality [32].

Interestingly, adherence to MD was also positively associated with sustainability awareness and purchasing behaviors. This agrees with the literature portraying the MD not only as a model of nutritional adequacy and chronic disease prevention but also as a culturally embedded and ecologically sustainable dietary pattern, particularly due to its emphasis on plant-based foods, low meat consumption, and seasonal eating habits [33].

These interconnected findings point toward the potential of integrated public health strategies that combine nutritional education with sustainability education. Educational interventions that simultaneously address the health benefits of plant-based diets and the environmental implications of food choices may be more effective in fostering behavioral change [34].

These findings underscore that although health-oriented diets may align with more sustainable practices, awareness alone may not be sufficient to drive behavior change toward environmentally sustainable eating.

The very strong correlation observed between knowledge of the health effects of fruits and vegetables and antioxidant knowledge (r = 0.869) suggests a substantial conceptual overlap between these constructs. Both measures likely capture a broader dimension of general nutrition knowledge rather than entirely distinct domains, which may partially explain the strength of the association observed. From a statistical perspective, such a high correlation raises the possibility of multicollinearity in the regression model, potentially affecting the stability and interpretability of the estimated coefficients. Although both variables were retained in the model due to their theoretical relevance and distinct conceptual framing, this issue should be acknowledged when interpreting the results. Future research should consider combining these measures into a composite index or applying dimensionality reduction techniques, such as factor analysis, to better distinguish underlying constructs. More refined and validated tools are needed to disentangle specific domains of nutrition knowledge, particularly when investigating their independent associations with behavioral outcomes.

This study has some limitations. First, the cross-sectional design does not allow for causal inferences, limiting the ability to determine the direction of the relationships observed. Second, the use of self-reported measures for diet adherence and knowledge may be subject to reporting biases. Third, the study population, while diverse, may not be entirely representative of the general population, as recruitment relied on chain mailing lists. Finally, further limitation of this study concerns the assessment of sustainability-related purchasing behavior. This variable was measured using a single, study-specific item rather than a validated, multidimensional instrument. As such, it may not fully capture the complexity of sustainability-related attitudes and behaviors, and the results should be interpreted with caution. Future research should employ validated and multidimensional instruments to provide a more comprehensive and reliable assessment of sustainability-oriented food choices.

## 5. Conclusions

In conclusion, while knowledge about healthy eating and antioxidants appears relatively widespread in the sample examined, the translation of this knowledge into sustainable consumption patterns remains limited. The correlations identified suggest that future interventions should not treat nutrition and sustainability as separate domains but rather address them in a unified framework. Promoting the MD may serve as an ideal entry point for advancing both individual health and collective environmental goals. Policymakers, educators, and health professionals should therefore consider integrated campaigns that foster both dietary and environmental literacy, paving the way toward healthier populations and a more sustainable food system.

Future research should also investigate consumers’ perceptions and knowledge of functional foods and dietary supplements derived from Mediterranean plant sources. Given the increasing commercialization of these products, understanding whether individuals recognize their bioactive and potential nutrigenomic properties, in addition to traditional whole foods, may help to better explain and address the gap between knowledge and actual dietary and sustainability-related behaviors.

## Figures and Tables

**Table 1:** Sample characteristics according to sex.

Variable	Total (*n* = 311)	Males (*n* = 131)	Females (*n* = 180)	*p*-Value
Age (years), mean ± SD	45.83 ± 15.16	44.43 ± 16.58	46.86 ± 14.44	0.164
Education level, ***n*** (%)				0.152
- Low	64 (20.6)	21 (16)	43 (23.9)	
- Medium	140 (45)	66 (50.4)	74 (41.1)	
- High	107 (34.4)	44 (33.6)	63 (35)	
BMI (kg/m^2^), mean ± SD	25.20 ± 3.64	25.76 ± 3.38	24.81 ± 3.78	0.022
Currently following a diet, ***n*** (%)	52 (17)	16 (12)	36 (20)	0.069

Values are presented as mean ± standard deviation (SD) for continuous variables and as number (percentage) for categorical variables. Differences between males and females were assessed using Student’s *t*-test for continuous variables and the chi-square test for categorical variables.

**Table 2:** Differences in main study variables according to sex, age group, and educational level.

Variable	Group	Mean ± SD	*p*-Value
Fruit and vegetable knowledge			
Sex	Male	0.83 ± 0.73	0.380
	Female	0.91 ± 0.76	
Age group	18–34	0.75 ± 0.79	0.062
	35–64	0.89 ± 0.73	
	≥65	1.11 ± 0.71	
Educational level	Low education	0.97 ± 0.72	0.423
	Medium education	0.82 ± 0.77	
	High education	0.90 ± 0.73	
Antioxidant knowledge			
Sex	Male	7.82 ± 4.12	0.819
	Female	7.93 ± 3.79	
Age group	18–34	7.19 ± 4.42	0.012
	35–64	7.85 ± 3.80	
	≥65	9.59 ± 3.07	
Educational level	Low education	8.41 ± 3.73	0.399
	Medium education	7.61 ± 3.91	
	High education	7.93 ± 4.07	
Medi-Lite score			
Sex	Male	9.24 ± 2.11	0.054
	Female	9.71 ± 2.12	
Age group	18–34	9.42 ± 2.05	0.207
	35–64	9.44 ± 2.01	
	≥65	10.12 ± 2.84	
Educational level	Low education	9.50 ± 1.81	0.067
	Medium education	9.24 ± 2.12	
	High education	9.87 ± 2.27	
Sustainability score			
Sex	Male	0.69 ± 0.96	0.826
	Female	0.67 ± 0.83	
Age group	18–34	0.68 ± 0.94	0.187
	35–64	0.64 ± 0.86	
	≥65	0.94 ± 0.92	
Educational level	Low education	0.73 ± 0.80	0.272
	Medium education	0.59 ± 0.86	
	High education	0.77 ± 0.96	

Values are presented as mean ± standard deviation (SD). *p*-values were calculated using independent samples *t*-tests for comparisons between males and females, and one-way ANOVA for comparisons across age groups and educational levels.

**Table 3:** Correlations (r and *p* values) between knowledge of health effects of fruits and vegetables, knowledge of antioxidants, adherence to MD and sustainable purchasing habits.

	Knowledge of Health Effects of Fruits and Vegetables	Antioxidant Knowledge	Medi-Lite Score	Sustainable Food Purchasing
Knowledge of Health Effects of Fruits and Vegetables	1	0.869 ** <0.001	0.076 0.193	0.678 ** <0.001
Antioxidant knowledge		1	0.100 0.084	0.663 ** <0.001
Medi-Lite Score			1	0.131 * 0.024
Sustainable Food Purchasing				1

Abbreviations: MD, Mediterranean diet. Pearson’s correlation coefficients (r) are reported. * *p* < 0.05; ** *p* < 0.01.

**Table 4:** Results of the logistic regression analysis with propensity to purchase sustainable foods as dependent variable.

Predictors	Odds Ratio	95% Confidence Interval		*p* Value
		Lower Limit	Upper Limit	
Age	1.354	0.674	2.721	0.394
Gender	1.096	0.555	2.163	0.792
Educational level	1.542	0.738	3.221	0.249
Knowledge of fruit and vegetable health effects	0.155	0.032	0.759	0.021
Antioxidant knowledge	0.030	0.003	0.251	0.001
Mediterranean diet adherence	0.618	0.310	1.231	0.171

Abbreviations: OR, odds ratio; CI, confidence interval. Results are from a logistic regression analysis. Statistical significance was set at *p* < 0.05.

## Data Availability

Dataset available on request from the authors.

## Abbreviations

The following abbreviations are used in this manuscript:

MD	Mediterranean Diet
BMI	Body mass index
